# Effects of dapagliflozin on cardiovascular outcomes in type 2 diabetes: Study protocol of a randomized controlled trial

**DOI:** 10.1097/MD.0000000000022660

**Published:** 2020-10-09

**Authors:** Dong Yin, Yuan Hui, Chuanhui Yang, Yi Xu

**Affiliations:** Department of endocrinology, Lianyungang Hospital affiliated to Xuzhou Medical University, Jiangsu, China.

**Keywords:** cardiovascular outcomes, dapagliflozin, randomized controlled trial, SGLT2, sodium–glucose cotransporter-2, study protocol, type 2 diabetic patients

## Abstract

**Background:**

Dapagliflozin, a novel inhibitor of renal sodium-glucose cotransporter 2, allows an insulin-independent approach to improve type 2 diabetes hyperglycemia. This current research is a double blinded, randomized, and prospective trial to determine the effect of dapagliflozin on cardiovascular outcomes in type 2 diabetes.

**Methods:**

This randomized controlled, double-blinded, single center trial is carried out according to the principles of Declaration of Helsinki. This present study was approved in institutional review committee of the Lianyungang Hospital affiliated to Xuzhou Medical University (LW-20200901001). All the patients received the informed consent. Diabetic patients were randomized equally to receive 28-week treatment with dapagliflozin or matching placebo. The major outcome of our current study was the change in the level of hemoglobin A1c (HbA1c) from the baseline to week 28. Secondary outcome measures contained the levels of fasting blood glucose, the mean change in seated systolic and diastolic blood pressure, body weight, and the mean change in calculated average daily insulin dose in patients treated with insulin at baseline, the other laboratory variables, and self-reported adverse events. The *P* < .05 was regarded as statistically significant.

**Results:**

We assumed that the dapagliflozin administration in patients with type 2 diabetes would reduce HbA1c, body weight, systolic blood pressure, and achieve the goal of glycemic control, without adversely impacting cardiovascular risk.

**Trial registration:**

This study protocol was registered in Research Registry (researchregistry5987).

## 1 Introduction

The cardiovascular disease (CVD) is still a direct and major cause of incidence rate and mortality among type 2 diabetic patients. In comparison with the non-diabetic people, the risk of cardiovascular disease is about 2 times that of non-diabetic patients.^[1]^ Chronic hyperglycemia can cause the complications of microvascular and macrovascular.^[2,3]^ In view of the increased health risk of complications and the clinical consequences,^[4]^ in order to effectively treat type 2 diabetes, it is necessary to control blood glucose, including strategies to treat the risk of hypertension, obesity as well as cardiovascular disease.^[5,6]^ The European Medicines Agency and the U.S. Food and Drug Administration have issued guidelines requiring the novel treatments for diabetes to exclude an unacceptable increase in the risk of cardiovascular disease.^[7]^ We need effective and safe treatment strategies to control the hyperglycemia and increase the clinical benefits.^[8,9]^

The familiarly utilized antidiabetic drugs (involving nonsulfonylurea secretagogues, sulfonylureas,^[10,11]^ and thiazolidinediones, metformin, glucagon-like peptide-1 analog,^[12]^ dipeptidyl peptidase-4 inhibitors as well as the α-glycosidase inhibitors) are insulin-dependent in their efficacy.^[13]^ In the development of type 2 diabetes, when the function of the islet β-cells decreases, they become less effective.^[14]^ Thiazolidinediones and sulfonylureas contribute to the weight gain and then in-depth worsen the insulin resistance.^[15]^ Not surprisingly, about two-thirds of type 2 diabetes patients fail to achieve their goal of blood glucose control under the regular treatment. In contrast, as the inhibitor of sodium glucose co-transporter 2 (SGLT2) with highly selective.^[16]^ Dapaglilizin is a kind of selective inhibitor of sodium–glucose cotransporter-2 (SGLT2), it reduces the levels of blood glucose via decreasing the reabsorption of glucose in kidney,^[17]^ it does not depend on the effect or secretion of insulin, leading to enhanced the urine glucose excretion, accompanied by caloric loss and osmotic diuresis.^[18]^ In the clinical trials, dapagliflozin has been well tolerated as the monotherapy or in combination with insulin, sulfonylureas and metformin.^[19,20]^ The action mechanism of dapaglilozin can affect many risk factors for cardiovascular disease, especially lowering the blood pressure, weight loss,^[21]^ decreasing the serum uric acid and albuminuria levels, lowering the waist circumference, and decreasing the inherent risk of hypoglycemia.^[22,23]^

This current research is a double blinded, randomized, and prospective trial to determine the influences of the dapagliflozin on the cardiovascular results of type 2 diabetes.^[24,25]^ We assumed that adding the dapagliflozin to the medication in the type 2 diabetes patients would reduce CVD risk and achieve the goal of glycemic control.^[26,27]^

## 2 Material and method

This randomized controlled, double-blinded, single center trial is implemented on the basis of the Declaration of Helsinki. This present study was approved in institutional review committee of the Lianyungang Hospital affiliated to Xuzhou Medical University (LW-20200901001). All the patients received the informed consent. It was also registered at the Research Registry (researchregistry5987).

### 2.1 Eligibility criteria

Inclusion criteria were the female or male patients with type 2 diabetes, and the age range is between 30 and 79 years, with an inadequate glycemic control, which were defined as HbA1c ≤9.5% and ≥6.6%. Subjects were required to register C-peptide≥ 0.27 nmol/l, the rate of estimated glomerular filtration less than 150 ml/minute/1.73 m^2^ and greater than 60 ml/minute/1.73 m^2^, the urinary albumin to the ratio of creatinine less than 300 mg/g, and the index of body mass ≤45.0 kg/m^2^, with an insufficient control of blood pressure, which was defined as the diastolic blood pressure less than 105 mm Hg and ≥80 mm Hg, and/or the systolic blood pressure less than 165 mm Hg and ≥130 mm Hg. Exclusion criteria included: a recent history of cardiovascular events, gestational diabetes, unstable renal disease, retinopathy, hepatic, or hematologic disease.

### 2.2 Randomization and blinding

**Figure 1:**
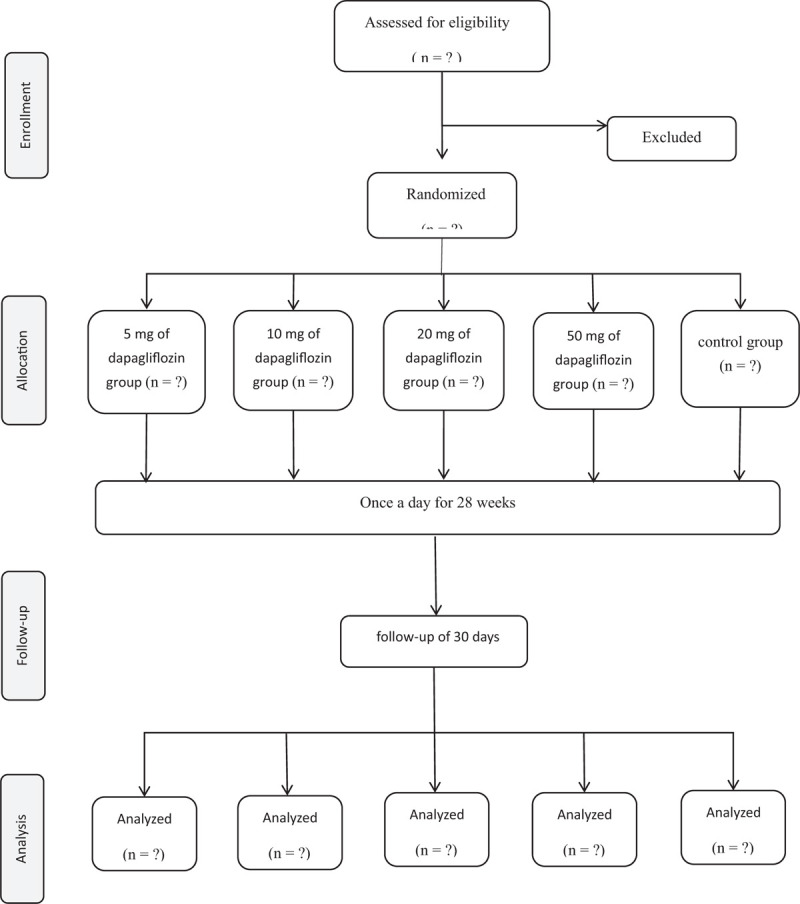
Consolidated standards of reporting trials statement flow diagram.

Each study group had an equal number of envelopes and the envelopes were produced via a research assistant through utilizing a computer-based random number generator who did not participate in any follow-up studies or contact with other study group members during the research period. He has prepared 150 identical, sealed, sequentially numbered, and opaque envelopes, with 30 envelopes containing the instructions for each group. These above envelopes have been submitted to the principal investigator for filing (Fig. 1).

### 2.3 Intervention and control

This is a placebo-controlled, double-blind, randomized trial, with a treatment of 28 weeks and a follow-up of 30 days. Then, the eligible patients in the introduction stage were randomly divided into 4 treatment groups with a blind manner (in a ratio of 1:1:1:1). Patients were equally given once-daily doses of dapagliflozin (5 mg, 10 mg, 20 mg, or 50 mg, respectively), or were given a matched placebo dose in combination with an established stable background treatment. Safety and efficacy were assessed at all study visits, the other laboratory variables, and self-reported adverse events. A value of *P* < .05 is considered as the significant in statistics.

### 2.4 Outcome measures

All the primary and critical secondary impact variables are pre-specified. The primary influencing variable was HbA1c change from baseline to week 28. At 28 weeks, the 5 primary secondary influencing variables involved:

1. the change of overall weight from baseline;
2. the change of 24-hour ratio of urine glucose to creatinine;
3. the average change of sitting diastolic and systolic blood pressure from baseline;
4. the change of fasting blood glucose from the baseline; and
5. the average change of calculated daily dose of insulin in the insulin treated patients at baseline.

The vital signs, laboratory parameters, and data on adverse events were collected for the evaluation of the long-term tolerability and safety of dapaglifrozin for 28 weeks.

### 2.5 Statistical analysis

With the Statistical Package for Social Sciences, the statistical analysis was implemented (SPSS for Windows, release 12.0; SPSS Inc., Chicago, IL). The statistical analysis was carried out via independent experts and it was not participated in the research program. The values of mean (range) and median were presented. Non-paired t-test was utilized for the numerical data of normal distribution. Non-parametric simulation was utilized where appropriate. The x^2^ test was utilized to compare the categorical variables. A value of *P* < .05 is considered as the significant in statistics.

## 3 Discussion

With the growth of age, type 2 diabetes patients usually treated through the antidiabetic drugs combining with other concomitant drugs to control comorbidities, for instance, obesity, hypertension and the cardiovascular disease. It is a recognized problem that type 2 diabetes patients possess an enhanced possibility of cardiovascular disease. Dapagliflozin is a kind of selective inhibitor of sodium-glucose co-transporter-2 that decreases the reabsorption of renal glucose with an insulin-independent mode. We attempted to determine the influence of dapagliflozin on lowering HbA1c, reducing systolic blood pressure and BW in patients at high risk for future cardiovascular disease events.

## Author contributions

**Conceptualization:** Dong Yin.

**Data curation:** Dong Yin.

**Formal analysis:** Dong Yin.

**Funding acquisition:** Yi Xu.

**Investigation:** Dong Yin.

**Methodology:** Yuan Hui.

**Project administration:** Yuan Hui, Yi Xu.

**Resources:** Yi Xu.

**Software:** Chuanhui Yang.

**Supervision:** Chuanhui Yang, Yi Xu.

**Validation:** Yuan Hui, Chuanhui Yang.

**Visualization:** Yuan Hui.

**Writing – original draft:** Dong Yin.

**Writing – review & editing:** Chuanhui Yang, Yi Xu.

## Abbreviations

Abbreviations: CVD = cardiovascular disease, HbA1c = hemoglobin A1c, SGLT2 = sodium glucose co-transporter 2.
